# The influence of δ-(Al,Fe)OOH on seismic heterogeneities in Earth’s lower mantle

**DOI:** 10.1038/s41598-021-91180-9

**Published:** 2021-06-08

**Authors:** Itaru Ohira, Jennifer M. Jackson, Wolfgang Sturhahn, Gregory J. Finkelstein, Takaaki Kawazoe, Thomas S. Toellner, Akio Suzuki, Eiji Ohtani

**Affiliations:** 1 Department of Earth Science, Graduate School of Science, Tohoku University, Sendai, 980-8578 Japan; 2 Seismological Laboratory, California Institute of Technology, Pasadena, CA 91125 USA; 3 Bayerisches Geoinstitut, University of Bayreuth, 95440 Bayreuth, Germany; 4 Department of Earth and Planetary Systems Science, Graduate School of Science, Hiroshima University, Higashi-Hiroshima, 739-8526 Japan; 5 Advanced Photon Source, Argonne National Laboratory, Argonne, IL 60439 USA; 6 Present Address: Department of Chemistry, Gakushuin University, 1-5-1, Mejiro, Toshima-ku, Tokyo, 171-8588 Japan; 7 Present Address: Department of Geosciences, Princeton University, Princeton, NJ 08544 USA

**Keywords:** Planetary science, Solid Earth sciences

## Abstract

The high-pressure phases of oxyhydroxides (δ-AlOOH, ε-FeOOH, and their solid solution), candidate components of subducted slabs, have wide stability fields, thus potentially influencing volatile circulation and dynamics in the Earth’s lower mantle. Here, we report the elastic wave velocities of δ-(Al,Fe)OOH (Fe/(Al + Fe) = 0.13, δ-Fe13) to 79 GPa, determined by nuclear resonant inelastic X-ray scattering. At pressures below 20 GPa, a softening of the phonon spectra is observed. With increasing pressure up to the Fe^3+^ spin crossover (~ 45 GPa), the Debye sound velocity (*v*_D_) increases. At higher pressures, the low spin δ-Fe13 is characterized by a pressure-invariant *v*_D_. Using the equation of state for the same sample, the shear-, compressional-, and bulk-velocities (*v*_S_, *v*_P_, and *v*_Φ_) are calculated and extrapolated to deep mantle conditions. The obtained velocity data show that δ-(Al,Fe)OOH may cause low-*v*_Φ_ and low-*v*_P_ anomalies in the shallow lower mantle. At deeper depths, we find that this hydrous phase reproduces the anti-correlation between *v*_S_ and *v*_Φ_ reported for the large low seismic velocity provinces, thus serving as a potential seismic signature of hydrous circulation in the lower mantle.

## Introduction

The circulation and distribution of “water” in the forms of hydrogen, hydroxyl, and molecular H_2_O in the Earth’s interior are important factors affecting the evolution and dynamics of the Earth’s interior^1–4^. Hydrous minerals store H_2_O in the order of 1–10 wt.% in their crystal structures, thus serving as large reservoirs of water even though their proportion in the mantle is relatively small.

An aluminum oxyhydroxide, δ-AlOOH, has a significantly wide stability field and therefore potentially plays a role as a hydrous reservoir in the Earth’s lower mantle. High pressure and high temperature experiments on δ-AlOOH have demonstrated that this phase is stable at 21–142 GPa and up to 2410 K^5–12^, comparable to the conditions from the lower regions of the mantle transition zone to the lowermost mantle. Experiments in natural-like multicomponent systems reveal the chemical composition, elasticity, and stability of δ-phase formed in the lower mantle environment, as discussed below^13–23^. In multicomponent systems, δ-AlOOH forms a solid solution with isostructural hydrous phases of ε-FeOOH and MgSiO_4_H_2_-Phase H^13–19^. However, the effect of incorporations of ε-phase (Fe) and Phase H (Mg, Si) on its stability appears to be limited because of strong partitioning of Al into the hydrous solid solution phase in lower mantle environments. In the MgO–Al_2_O_3_–Fe_2_O_3_–SiO_2_–H_2_O (60 mol% MgSiO_3_–30 mol% Al_2_O_3_–10 mol% Fe_2_O_3_ containing ~ 7 wt.% H_2_O) system, the hydrous solid solution (δ-phase) coexists with bridgmanite, its high-pressure polymorph (post-perovskite), or both at 104–126 GPa and 1750–2500 K^18^. The chemical analyses of the run products at 117 GPa and 2050 K in this system showed that the composition of the synthesized δ-phase was (Mg_0.03(2)_Si_0.07(3)_Al_0.81(4)_Fe_0.09(3)_)OOH^18^. Such a Al-rich composition is similar to the hydrous phase (Mg_0.11_Al_0.63_Si_0.2_Fe_0.03_)OOH, formed in a natural basalt system at 25–26 GPa and 1273–1473 K^17^ and closer to AlOOH than FeOOH and MgSiO_4_H_2_. In contrast to the stability, the elasticity of δ-AlOOH might be altered largely due to a presence of modest amounts of Fe. δ-AlOOH, ε-FeOOH, and their solid solution (hereafter, δ-(Al,Fe)OOH) have a *Pnnm* structure with a symmetric hydrogen bond at lower mantle pressures^20,21–26^. Substitution of Fe into the Al site of the δ-phase causes an increase in density^22^. In addition, the high-spin–low-spin transition of Fe^3+^ in δ-(Al,Fe)OOH at 32–40 GPa, corresponding to shallow lower mantle depths, causes a softening of the isothermal bulk modulus, which consequently decreases *v*_Φ_^22^. Moreover, a theoretical study proposed a negative correlation of pressure (*P*)–shear modulus (*μ*) for low-spin ε-FeOOH, which causes a decrease of *v*_S_/*v*_Φ_ with increasing pressure^26^. These studies point to the possibility that the solid solution may in part be responsible for regionally seismic heterogeneities observed in the lower mantle^27,28^.

As discussed above, δ-(Al,Fe)OOH is likely stable in subducted slab materials throughout the lower mantle^18^, yet the possible effects on the seismic wave velocities of a phase assemblage containing the δ-phase are not well constrained. In this study, we report the elastic wave velocities of the δ-(Al_,_Fe)OOH phases having an Fe/(Al + Fe) ratio of ~ 0.13 determined by nuclear resonant inelastic X-ray scattering (NRIXS). The Fe/(Al + Fe) ratio of our sample is within the rations of δ-phase formed in the MgO–Al_2_O_3_–Fe_2_O_3_–SiO_2_–H_2_O system that simplifies natural basaltic compositions (Fe/(Al + Fe) = 0.10–0.17)^18^. By combining our results from NRIXS with the equation of state^22^, we discuss the potential relationships between δ-(Al,Fe)OOH and seismic anomalies in the lower mantle.

## Results

### Determination of elastic wave velocities

**Figure 1:**
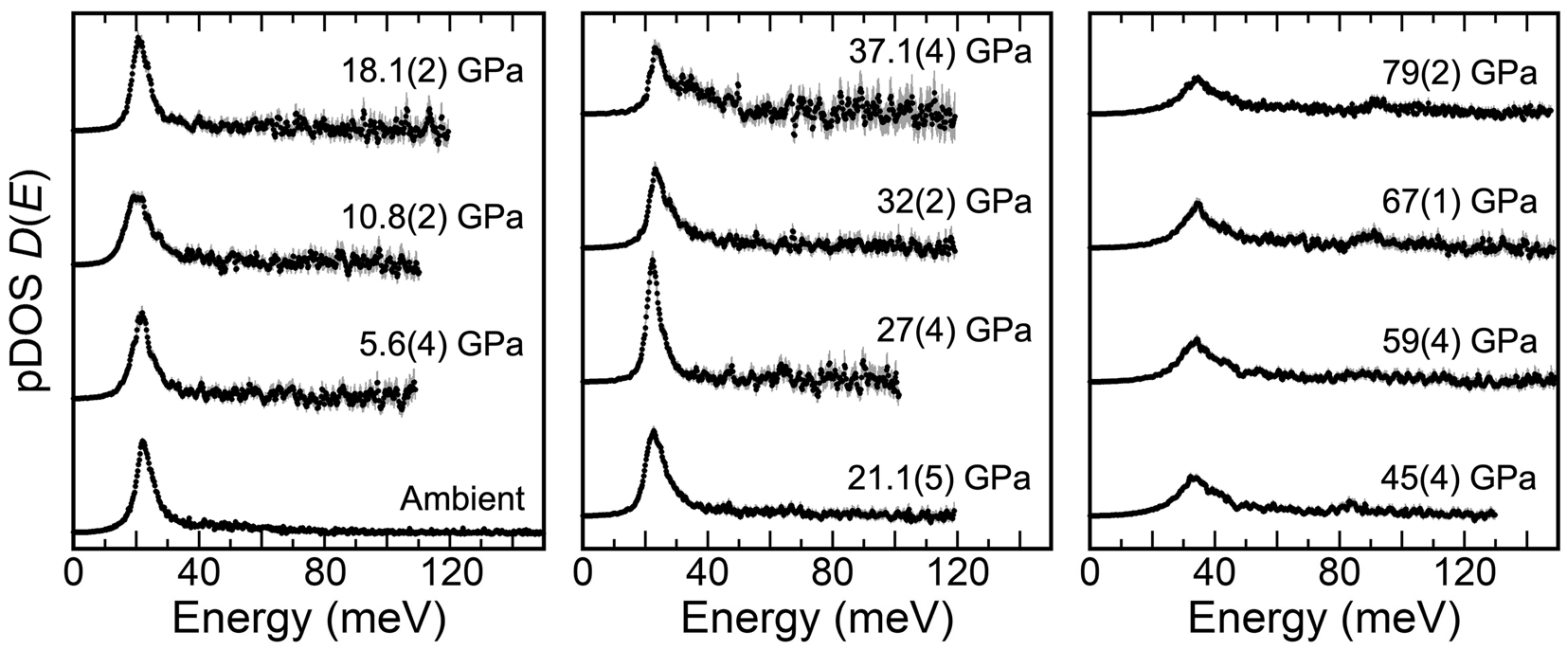
The partial projected phonon density of states (pDOS) of δ-(Al_0.873_^57^Fe_0.127_)OOH (δ-Fe13-r1) and δ-(Al_0.867_^57^Fe_0.133_)OOH (δ-Fe13-r2). The errors are indicated by shaded bars, and uncertainty in pressure is given in parentheses.

**Figure 2:**
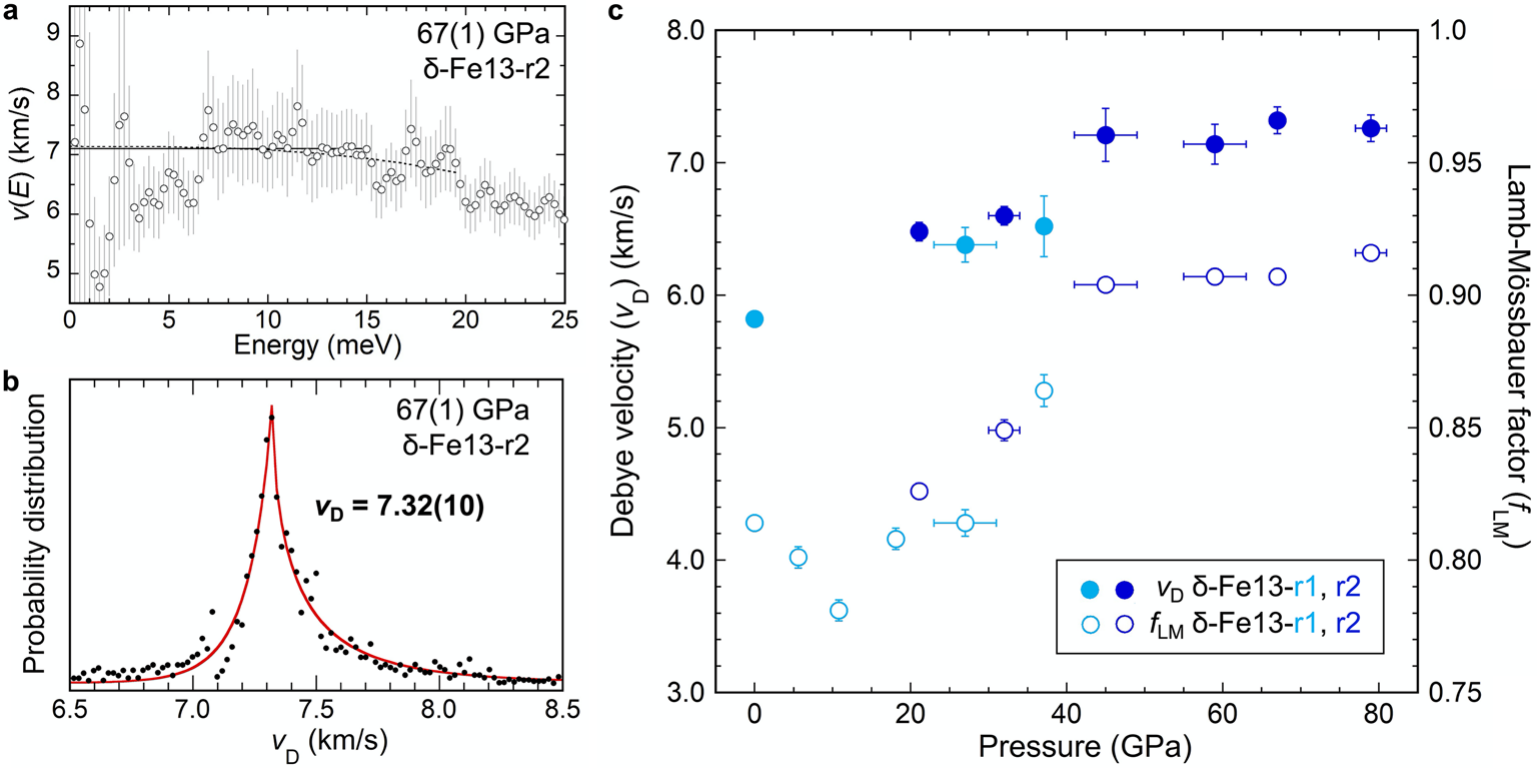
**(a)** An example of the Debye sound velocity determination of δ-Fe13 from the pDOS determined at 67 GPa. Examples of two typical phonon dispersion models that are traditionally used for constraining the Debye sound velocity (*v*_D;_ *v*(*E*) at E = 0) are plotted: the Debye-like model (horizontal black solid line) and the power law model (Eq. 1, black dotted line). The open circles are the pDOS data plotted according to Eq. (2). We note that the fit models plotted here are purely demonstrative and do not directly reflect our reported Debye velocities. Families of these models are considered, fit to the data, and plotted as probability distributions (ref.^33^) of the *v*_D_. **(b)** The energy ranges applied in the PDF analysis at 67 GPa fall within, but are not limited to, the *E*_min_ and *E*_max_ values given in Table S1. See text for a detailed description of our Debye velocity determination method. **(c)** The correlation of *v*_D_ (solid circle) and Lamb–Mössbauer factor (*f*_LM_, open circle) of the δ-Fe13 samples; *v*_D_ values account for the ^57^Fe enrichment of the samples.

NRIXS spectra of the two δ-(Al_0.873_^57^Fe_0.127_)OOH (δ-Fe13-r1) and δ-(Al_0.867_^57^Fe_0.133_)OOH (δ-Fe13-r2) samples were obtained up to 79 GPa (Fig. S1). The ^57^Fe-partial projected phonon density of state (pDOS) was subsequently extracted from each NRIXS spectrum using the PHOENIX software^29^ (Fig. 1). Generally, vibrational peak positions in the pDOS for minerals steadily shift to higher energies with compression^30,31^. However, the peak positions of pDOS of δ-Fe13 shift to lower energies with increasing pressure from ambient pressure to 10.8 GPa, above which they shift to higher energies (Fig. 1). This pressure at which the peak position of pDOS of δ-Fe13 reaches the lowest energy is very close to that of the structural transition from *P*2_1_*nm* with asymmetric hydrogen bonds to *Pnnm* with symmetric hydrogen bonds inferred from XRD experiments on δ-(Al,Fe)OOH (Fe/(Al + Fe) = 0.047(10) (hereafter, δ-Fe5) and 0.123(2) (δ-Fe12))^22^. Static calculations for pure δ-AlOOH predicted that phonon-softening occurred as a result of this transition involving hydrogen bonding^32^. Through this transition, the lowest and highest *A*_1_ and *B*_2_ optic modes soften and the OH stretching frequency approaches nil^32^. The Raman spectra of polycrystalline δ-AlOOH show broadening or disappearance of *B*_2_ mode of *P*2_1_*nm* and the appearance of *A*_g_ mode of *Pnnm* near 5.6 GPa^20^. Therefore, the shifting of the pDOS to lower energies below about 10 GPa and the trend observed in the Lamb-Mössbauer factor (*f*_LM_) of δ-Fe13 (Fig. 2) are likely related to this transition. Due to the likelihood that one or more optical branches cross into the accessible low-energy regime in this low-pressure range (~ 2 < E (meV) <  ~ 15), the assumption that the low-energy region of the pDOS is occupied purely by acoustic modes breaks down. Thus, we restrict our determination of the Debye sound velocity (*v*_D_) to ambient pressure and pressures higher than 20 GPa.

A representative Debye sound velocity determination is shown in Fig. 2a. In this figure, two different models for phonon dispersion are plotted; one model is a ‘Debye-like’ model that plots as a constant value, and the other is an empirical power law model for phonon dispersion defined as Eq. 1:

1 $${{v}}({{E}}) = {{v}}_{\text{D}} \, \left[{1} \, - {\left({{E}}/{{A}}_{1}\right)}^{{{A}}_{2}}\right],$$

where *A*_1_ and *A*_2_ are constants that are optimized by a standard least squares fitting process. In this power law model, *v*_D_ is defined as *v*(0) in this profile. Note that ‘Debye-like’ dispersion is accommodated in Eq. (1).

However, low-count rate experiments such as these pose challenges to determining *v*_D_. For example, the values determined from these two dispersion models may be sensitive to the fitting energy range for pDOS and the phonon dispersion model, especially if there is scatter in the data. To place better constraints on *v*_D_ without relying on the choice of an energy fit range, the analysis of a probability distribution function (PDF) of *v*_D_ proposed by Morrison et al. (ref.^33^) was applied in this study and an example at 67 GPa is shown in Fig. 2. The PDF method accounts for hundreds of physically reasonable fit ranges and phonon dispersion behavior in the determination of *v*_D_ (Eq. (1)), and therefore places a more reasonable constraint on *v*_D_ and its uncertainty than would be calculated only from a single energy range^33^. The details of the PDFs, including the energy ranges considered and bin size are provided in Table S1. The reported *v*_D_ values and their uncertainty are the peak positions and full width at half maximum of fitting an asymmetric function to the PDFs, respectively (Figs. 2b, S2; Tables S1 and S2). The ^57^Fe concentration of the sample was used in the determination of *v*_D_.

The elastic wave velocities, *v*_P_, *v*_S_, and *v*_Φ_, were calculated from *v*_D_ using Eqs. (3) and (4) (see Methods). The isothermal bulk modulus (*K*_*T*_) and density (*ρ*) were calculated from the equation of state (EoS) parameters of δ-(Al_0.877_^57^Fe_0.123_)OOH (hereafter δ-Fe12, ref.^22^) using the MINUTI software version 2.1.0^29^. The 2nd-order Birch-Murnaghan EoS parameters of δ-Fe12 with asymmetric (ordered) hydrogen bonds and high-spin state (*K*_*T*0_ = 147 ± 1 GPa, *K*_*T*_^′^ = 4, *V*_0_ = 57.85 ± 0.02 Å^3^)^22^ were applied to the calculation of *v*_D_ at ambient conditions, and the spin crossover EoS parameters for δ-Fe12 with symmetric (disordered) hydrogen bonds (high-spin state, *K*_*T*0_ = 155 ± 22 GPa, *K*_*T*_^′^ = 8 ± 2, *V*_0_ = 57.5 ± 0.3 Å^3^; low-spin state, *K*_*T*0_ = 241 ± 14 GPa, *K*_*T*_^′^ = 4, *V*_0_ = 55.2 ± 0.4 Å^3^)^22^ were used to calculate the *v*_D_ at pressures higher than 20 GPa. We first determined the Debye velocities and density with the experimental mass (i.e., 96.64% ^57^Fe-enriched molecular mass of δ-Fe13), and then converted these to values corresponding to natural isotopic enrichment by using Eqs. (8) and (9), which are used in the geophysical discussion. The *K*_*T*_ obtained from the EoSs was converted to the adiabatic bulk modulus (*K*_*S*_) by using the Grüneisen parameter (*γ*_0_ = 0.64 ± 0.05), the exponent of its volume dependence (*q* = 1.8 ± 0.3) and Debye temperature (Θ_0_ = 1485 ± 300 K) of δ-AlOOH reported by ref.^11^.

### Behavior of elastic wave velocities under compression

The *v*_P_, *v*_S_, and *v*_Φ_ values of the two δ-Fe13 samples are summarized in Fig. 3 and Table S2. We make the following observations and compare with previous results. The concentration of iron in our sample is three times higher than the δ-(Al_0.956_Fe_0.044_)OOH polycrystalline sample (hereafter, δ-Fe4) measured using Brillouin light spectroscopy, thus when compared with the Al end-member values and the δ-Fe4 sample, our ambient pressure *v*_P_ and *v*_S_ values are consistent with this trend. The *v*_S_ of δ-Fe13 increases up to the pressure conditions of the Fe^3+^ spin transition^22^, although the slope is shallower than that reported for δ-Fe4^23^ and δ-AlOOH^20^ (Fig. 3). The lower *v*_S_ values we report are reasonably explained by the difference in Fe concentration, as noted above. However, other factors such as preferred orientation could offer explanations. The NRIXS measurements used crushed single grains of a more iron-rich composition and the Brillouin scattering measurements used a potentially finer-grained polycrystalline iron-poor sample^23^. These differences might enhance the effect of orientation and/or intergranular stresses on measured data in the vicinity of phase transitions. Interestingly and unlike other materials undergoing a spin crossover, the polycrystalline Brillouin scattering results do not show any softening of *v*_P_ near this transition^23^. At 32 and 37.1 GPa, both *v*_P_ and *v*_Φ_ decrease due to the reduction of *K* influenced by Fe^3+^ spin transition^22^ (Fig. 3a,c and Table S2). A decrease in *K* as a result of the spin transition is also observed in other low-Fe content minerals (e.g., ferropericlase^34,35^). The degree of reduction of *v*_P_ and *v*_Φ_ of δ-Fe13 due to the spin transition is significant. Specifically, the *v*_P_ and *v*_Φ_ values at 37.1 GPa are decreased by 13% and 23% compared to those at 27 GPa. After the completion of the Fe^3+^ spin transition (45 GPa and higher pressures), both *v*_P_ and *v*_Φ_ increase steadily with compression (Fig. 3a,c).

**Figure﻿ ﻿3:**
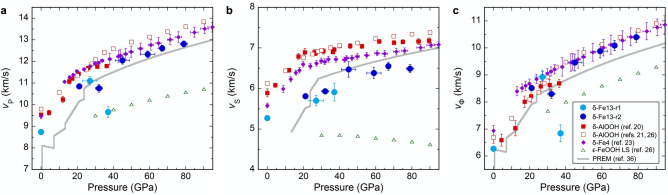
The elastic wave velocities of the δ-Fe13 samples computed using the natural abundance of Fe: **(a)**, *v*_P_, **(b)**, *v*_S_, **(c)** *v*_Φ_. Elastic wave velocities of other end-membered hydrous phases: δ-AlOOH^20,21,26^, δ-(Al_0.956_Fe_0.044_)OOH (δ-Fe4)^23^, and ε-FeOOH with low spin state of Fe^3+^^26^ are also shown. PREM is shown by gray lines^36^. The values indicated by solid and open symbols were obtained from room temperature experiments and calculations at 0 K, respectively. Abrupt reductions of *v*_P_ and *v*_Φ_ for the δ-Fe13 samples at 32 GPa and 37.1 GPa are due to the spin transition of Fe^3+^^22^. The numerical values for the data of δ-Fe13 samples in this figure are available in Table S2.

Above 45 GPa, the pressure–velocity profiles of δ-Fe13 do not show any abrupt changes (Fig. 3). Pressure dependencies of velocities, however, are significantly different among *v*_P_, *v*_S_, and *v*_Φ_. The *v*_P_ and *v*_Φ_ exhibit relatively large gradients with pressure, similar to the Preliminary Reference Earth Model (PREM)^36^. In contrast, the *v*_S_ values are essentially invariant with respect to pressure, such that the values of *v*_S_ determined in this study above 45 GPa are equal within the associated errors (6.38–6.55 ± 0.10–0.18 km/s). This leads to a weak pressure dependence of the shear modulus (*μ*). The *μ* increases from 190 to 206 GPa between 45 and 79 GPa, which is within the range of errors: ± 0.06 to ± 0.11 (Table S2).

## Discussion

The spin transition of Fe^3+^ in the δ-Fe13 samples results in a high *v*_S_/*v*_Φ_ (0.86 at 37.1 GPa) (Table S2), which is ~ 19% higher than PREM at this corresponding depth (~ 970 km). The spin transition of Fe^3+^ in the octahedral site is also observed in the Fe-bearing NAL phase^37^. However, pressure condition and width of spin crossover involving volume collapse are slightly lower and narrower in the δ-Fe5 and δ-Fe12 (32–40 GPa)^22^ than the (Na_0.71_Mg_2.05_Fe^2+^_0.09_Al_4.62_Fe^3+^_0.17_Si_1.16_O_12_) NAL phase (33–47 GPa)^37^. A computational study by ref.^38^ shows that the spin transition pressure of Fe^3+^ in the octahedral site of the NAL phase is ~ 40 GPa, and it remains mostly invariant to temperature and the width moderately increases with temperature. At 300 K, the isothermal bulk modulus of the Fe^3+^-bearing NAL phase (NaMg_2_(Al_4.67_SiFe_0.33_)O_12_) in the pressure range of spin crossover is up to ~ 25% lower than the bulk modulus-pressure trend expected without a spin transition^38^. The reduction of bulk modulus is limited to ~ 8% at 1200 K^38^, the estimated temperature of a slab penetrated into the top of the lower mantle^39,40^, resulting in the ~ 4% reduction of *v*_Φ_. At 300 K, the isothermal bulk modulus of δ-Fe13 throughout the spin crossover is up to ~ 55% lower than the bulk modulus-pressure trend expected without a spin transition, resulting in the ~ 33% reduction of *v*_Φ_. This would imply that for δ-Fe13 at about 1200 K the pressure of the spin-crossover is likely to be unchanged from that measured at 300 K and the resultant softening of the bulk modulus remains appreciable compared to the Fe^3+^-bearing NAL phase^37^. This behavior is different than the Fe^2+^ spin crossover in ferropericlase, in that although ferropericlase exhibits elastic softening, the transition occurs at deeper depths^34,35^ and exhibits a stronger temperature dependence^41^ than that for the Fe^3+^-bearing phases discussed above. It is also different from that of Fe^3+^-bearing bridgmanite^42^ and stishovite^43^.

Seismic observations in this region just below the transition zone (< 1000 km in depth) suggest a range of anomalies^44^, inferred to be correlated with the presence or stagnation of slab debris^45^. Therefore, the high *v*_S_/*v*_Φ_ in the spin crossover region of δ-Fe13 (and NAL) could be related to these seismic anomalies, especially in regions of relatively lower temperatures, such as those calculated for subducted slabs. Further studies on the temperature dependence of these properties across the spin transition would help quantify this effect.

Above 45 GPa, the gradients of *v*_P_ and *v*_Φ_ (d*v*_P_/d*P* and d*v*_Φ_/d*P*) of δ-Fe13 are close to those of PREM (Fig. 3a,c). By contrast, that of *v*_S_ (d*v*_S_/d*P*) is practically constant above 45 GPa (Fig. 3b). To discuss velocity relations between δ-Fe13 and PREM at deep-lower mantle pressure conditions, we calculated the elastic wave velocities for pressures higher than 80 GPa by using the following extrapolation procedure. First, we determined the elastic wave velocities at room temperature (Table S2). The *v*_P_ values were extrapolated using a linear relationship (Birch’s law^46^) between the *ρ* calculated from the thermal EoS (see ‘Determination of elastic wave velocities’ section) and the measured *v*_P_ of low-spin δ-Fe13 at 45 GPa, 59 GPa, 67 GPa, and 79 GPa (Table S2). The *v*_Φ_ was calculated from those thermal EoS. The *v*_S_ was then calculated from the extrapolated *v*_P_ and the calculated *v*_Φ_ by using the Eqs. (5)–(7).

**Figure 4:**
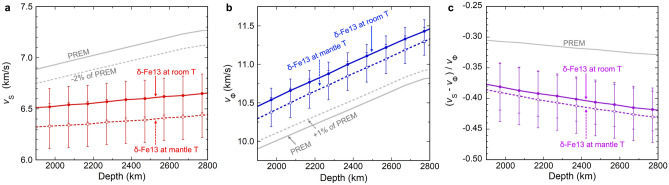
The depth–velocity profiles of δ-Fe13 and PREM^36^: **(a)** *v*_S_, **(b)** *v*_Φ_, and **(c)** (*v*_S_ − *v*_Φ_)/*v*_Φ_. The plots of δ-Fe13 at room T (solid circle) and mantle T (open circle) indicate the velocities of δ-Fe13 at room temperature and along an average mantle geotherm^48^, respectively. The calculation procedure of *v*_S_ and *v*_Φ_ of δ-Fe13 under these conditions is explained in the main text. The velocities of δ-Fe13 shown in this figure are determined using the density of δ-Fe13-r2 with natural mass of Fe. The dotted gray lines indicating − 2% of *v*_S_ and + 1% of *v*_Φ_ relative to PREM (solid gray lines) are regarded as reasonable perturbations for each respective velocity within the LLSVPs, based on the SB10L18 model^27^. The degree of anti-correlation between *v*_S_ and *v*_Φ_ in δ-Fe13 is considered to be larger than the maximum anti-correlation observed in the LLSVPs. Estimated uncertainties for δ-Fe13 are shown by the vertical bars. The numerical values for the data shown in this figure are available in Table S3.

The *v*_S_ of δ-Fe13 at room temperature is 5% slower than PREM at 1871 km in depth, and then becomes 9% slower than PREM at 2771 km in depth, 120 km above the core–mantle boundary. The difference of *v*_S_ between δ-Fe13 and PREM becomes larger with depth because of the very week pressure dependence of shear modulus of δ-Fe13 (Fig. 4). On the other hand, *v*_P_ and *v*_Φ_ at room temperature are 1% and 6% faster than PREM at 1871–2771 km in depth, respectively (Fig. 4, Table S3). The difference of *v*_S_ and *v*_Φ_ between δ-Fe13 and PREM suggests that an incorporation of 17 vol.% δ-Fe13 into PREM (representing average mantle) can account for − 0.9 to − 1.5% anomaly for *v*_S_ and + 1% anomaly for *v*_Φ_ in this depth range if the temperature dependences of velocities of δ-Fe13 are not considered.

Such an anti-correlation between the *v*_S_ and *v*_Φ_ has been reported for the large low-shear-velocity provinces (LLSVPs) in the depth range of 2000 km down to the core-mantle boundary, beneath the Pacific Ocean and the region spanning underneath the Atlantic Ocean to the western and southern part of the African continent^27,28^. It has been suggested that the LLSVPs are surrounded by downwellings, driven by slab subduction^47^. Therefore, if slabs contain a hydrous δ-phase and these slabs are transported into the deep-lower mantle, then slab debris containing the δ-phase could accumulate in the LLSVPs over geologic time, although it is not clear what the exact thermal state of this assemblage would be. To compare the elastic wave velocities of δ-Fe13 and PREM along an average mantle geotherm^48^, we apply the temperature dependence of *v*_S_, which was reported for MgSiO_3_-bridgmanite at 100 GPa (i.e., d*v*_S_/d*T* =  − 1 × 10^–4^ (km/s)/K) at 100 GPa^49^. Another study demonstrated that the d*v*_S_/d*T* of newly discovered FeO_2_H_x_ P-phase is also approximately − 1 × 10^–4^ (km/s)/K, at 133 GPa^50^. The *v*_Φ_ along the mantle geotherm was calculated from the thermal EoS parameters used in the calculation of *v*_Φ_ at room temperature, and *v*_P_ was subsequently calculated from the extrapolated *v*_S_ and *v*_Φ_ along an average mantle geotherm by using the Eqs. (5)–(7). In this case, the *v*_P_ and *v*_S_ of δ-Fe13 along the mantle geotherm^48^ are 1% and 8 to 11% slower than PREM in the depth range of 1871–2771 km, respectively, whereas *v*_Φ_ of δ-Fe13 is 4 to 5% faster than PREM (Fig. 4, Table S3). The anti-correlation between *v*_S_ and *v*_Φ_ results in ~ 30% lower (*v*_S_ − *v*_Φ_)/*v*_Φ_ ratio for δ-Fe13, compared with the PREM model (Fig. 4).

In this discussion, we used *γ*_0_ = 0.64 (with *q* = 1.8 and Θ_0_ = 1485 K reported for δ-AlOOH^11^). However, the effect of spin transition on the Grüneisen parameter of δ-Fe13 has not been investigated. To address the uncertainty of the Grüneisen parameter in our extrapolations, we tested a value of 1.28 as *γ*_0_ which is twice as large as the *γ*_0_ used in the present discussion (0.64)_._ The doubled value of the Grüneisen parameter decreases the *v*_Φ_, the most sensitive parameter of all three velocities (*v*_P_, *v*_S_, and *v*_Φ_) to changes in the Grüneisen parameter, by $\le$ 0.12 km/s (1.2% reduction) at the pressure and temperature conditions of 1871–2771 km depth, which are smaller than the errors of the extrapolated velocities (Fig. 4 and Table S3). Such a significant change in the Grüneisen parameter is unlikely for δ-Fe13. For example, a recent calculation for (Mg_0.75_Fe_0.25_)O ferropericlase showed that at 120 GPa the Grüneisen parameters of high- and low-spin states were 1.94 and 2.05 at 1400 K, respectively^51^. As discussed above, δ-Fe13 is primarily in the low-spin state at 80 GPa under elevated temperatures. If the difference in the Grüneisen parameter between high-spin and low-spin δ-Fe13 is also ~ 0.1, the velocity reductions are smaller than the example we tested above. To place a better constraint on the Grüneisen parameter of Fe-bearing δ-phase through the spin transition, high-temperature experiments and computational studies are required.

When assuming an incorporation of 7 vol.% of δ-Fe13 into the average mantle (PREM) at 1871–2771 km depth, − 0.6 to − 0.8 ± 0.2% anomaly of *v*_S_ and + 0.3 ± 0.2% anomaly of *v*_Φ_ would be generated, which are likely regarded as typical anomalies where the edge regions of LLSVPs have been sufficiently sampled. Although this discussion involves simplifications for the petrology and significant extrapolations, as well as noting that the magnitudes of anomalies in the seismic wave velocity and density depend on the seismic models^52,53^, our new data suggests that the presence of δ-(Al,Fe)OOH can produce seismic heterogeneities in the deep lower mantle. The total mass of LLSVPs is estimated to be 1.91 wt.% of the entire mantle^54^. On the other hand, the amount of recycled oceanic crust that entered and stayed in the lower mantle since 4 Gyr is estimated as ~ 3–5 wt.% of the present mantle^55,56^, which is at least 1.5 times larger than the mass of LLSVPs. It has been suggested that about one ocean mass of water (0.037 wt.% of the mantle) has been transported via slabs to the deep mantle over the age of Earth^57^. If this amount of H_2_O is completely stored in LLSVPs, a hypothetical and unlikely end-member scenario, via a dense oxyhydroxide like the delta phase, the H_2_O content of LLSVPs is calculated to be 1.9 wt.%. This scenario suggests that a hydrous mafic system, such as the system examined in ref.^17^ where the Al-rich Phase H (i.e., the Mg, Si, Fe-bearing δ-phase) could accumulate in LLSVPs. If this 1.9 wt.% H_2_O in the LLSVPs is completely stored in δ-Fe13, then this hydrous phase accounts for 12 vol.% of the LLSVP assemblage. This amount is larger than the 7 vol.% concentration of δ-Fe13 that can reproduce the typical anomalies at the edge regions of LLSVPs, as discussed above. The incorporation of 12 vol.% δ-Fe13 into “PREM” at 1871–2771 km depth can cause − 1.0 to − 1.4 ± 0.3% anomaly for *v*_S_ and + 0.5 to + 0.6 ± 0.3% anomaly for *v*_Φ_, which are comparable to the anomalies reported in the central regions of LLSVPs^27^. If the temperature is higher than the phase stability, then the δ-phase will dissociate. If these conditions are met, then it is possible that released hydroxyl migrates upward and hydrates regions at lower temperatures^58^, although much work is still needed to understand the phase relations of such processes.

Our elastic wave velocity results shed a new light on lower mantle seismic observations. At shallow lower mantle depths (800–900 km), δ-(Al,Fe)OOH exhibits low-*v*_Φ_ and *v*_P_ due to the spin transition, which may explain some seismic anomalies in this depth range. At deeper depths, the elastic behavior of δ-(Al,Fe)OOH is unlike typical lower mantle phases, and we discuss its relevance to LLSVP observations. Compositional differences between the LLSVPs and the surrounding mantle have been proposed as an origin of LLSVPs’ seismic structure^47, 59–61^. Our study proposes that if δ-(Al,Fe)OOH is formed by the reaction between oceanic crust and water^17,18^, and accumulates in deep local regions such as the edge regions of LLSVPs, those regions can reproduce the seismically observed negative anomaly of *v*_S_ and the positive anomaly of *v*_Φ_.

## Methods

Single crystals of Fe-bearing δ-phase were synthesized by the high-pressure hydrothermal method using a 1000-ton multi anvil apparatus (Hymag installed at Bayerisches Geoinstitut, University of Bayreuth), as previously reported by ref.^62^. The starting material of synthesis was a mixture of reagent-grade Al(OH)_3_ (Rare Metallic Co., Ltd.) and Fe_2_O_3_ (96.64% ^57^Fe, ISOFLEX) powders with Fe/(Al + Fe) = 0.15 in molar. This powder mixture packed into a welded Au_80_Pd_20_ capsule was hold at 21 GPa and 1470 K for 4 h, followed by rapid quenching. From the aggregates of synthesized crystals, two grains of δ-(Al_0.873(3)_^57^Fe_0.127(3)_)OOH with dimensions of ~ 50 × 60 × 10 µm (Run 1, δ-Fe13-r1) and δ-(Al_0.867(3)_^57^Fe_0.133(3)_)OOH with dimensions of ~ 40 × 50 × 20 µm (Run 2, δ-Fe13-r2) were selected for this study. The chemical compositions of the two samples were determined using an electron microprobe operating in wavelength-dispersive mode (JEOL, JXA-8800 installed at Tohoku University) operated at 15 kV and 10 nA. The numbers in parentheses in the chemical compositions indicate one standard deviation of ten measurement data for each sample. The H_2_O contents were calculated from oxide deficit in total mass in the microprobe analyses, as reported in ref.^62^. The weight deficit analyses indicated that δ-Fe13-r1 and δ-Fe13-r2 contain 2.3 wt.% and 2.9 wt.% excess H_2_O, respectively, which are included in calculation of the density of the two samples.

High-pressure NRIXS experiments were performed using panoramic diamond anvil cells (pDACs). Beryllium disks are used as gaskets. The disks were pre-indented to thicknesses of 40–50 μm, and then 210 μm or 165 μm (in diameter) holes were drilled in the center of gaskets. The former and latter gaskets were used for the pDACs with 400 μm-culet anvils (Run 1, δ-Fe13-r1) and 300 μm-culet anvils (Run 2, δ-Fe13-r2), respectively. A mixture of 10–20 μm thick boron epoxy (amorphous boron powder:epoxy = 4:1 by weight) is attached to the gasket holes, which can stabilize the sample room at high pressures. We put the sample in the center of gasket hole and two ruby spheres beside the sample as pressure markers, and then loaded a compressed neon gas as a pressure-transmitting medium into the sample chamber of pDACs. The gas-loading was conducted at the Seismological Laboratory, California Institute of Technology. The cell design for the pDAC using 300 μm-culet anvils is same as that used in the previous synchrotron Mössbauer spectroscopy experiments by Ref.^22^.

We conducted NRIXS measurements at room temperature and high pressures up to 79 GPa at Sector 3-ID-B at the Advanced Photon Source. During the measurements, the storage ring was operated in 24-bunch top-up mode. Each bunch was separated by 153 ns. Incoherent inelastic X-ray scattering was detected with three avalanche photodiode detectors (APDs) positioned radially around the pDACs. In addition to the three APDs, another APD located at a downstream parallel to X-ray path was used for collecting forward elastic scattering, which helps constrain the resolution function. The energy of incident X-ray was tuned around 14.4125 keV, the energy of nuclear resonance of ^57^Fe, by using a high-resolution monochrometer^63^ with a typical energy resolution (full width at half maximum at zero energy transfer) measured to be about 1.1 meV. The details of the setup of NRIXS experiments at Sector 3-ID-B are presented elsewhere^33,63,64^. Here, we note a brief summary of conditions of the NRIXS experiments. Energy scans were performed over the ranges of − 100 to + 150 meV (ambient conditions in air), − 80 to + 110 meV (at 5.6 and 10.8 GPa), − 80 to + 120 meV (18.1, 21.1, 27, 32 and 37.1 GPa) and − 80 to + 150 meV (45, 59, 67 and 79 GPa), with an energy step-size of 0.25 meV. The pressure at each compression point was determined using the ruby fluorescence method^65^. The ruby fluorescence spectra were obtained before and after collecting NRIXS spectra at each pressure point using an off-line Raman spectrometer at the Sector 3-ID-B. Pressure errors arise from the standard deviation between the four ruby measurements obtained before and after NRIXS measurements for two ruby spheres, and the error of ruby scale^66^. The raw NRIXS data were analyzed by using the version 3.0.0 PHOENIX software package (www.NRIXS.com) to obtain the partial phonon density of states (pDOS), *v*_D_, and the Lamb-Mössbauer factor^33,67,68^. From the low-energy region of pDOS, *v*_*D*_ is calculated from the following equation:

2 $${{v}}({{E}}) = {\left\{\frac{{{m}}{{E}}^{2}}{{2}{\uppi}^{2}{{\hbar}}^{3}{\rho D}({{E}})}\right\}}^{\frac{1}{{3}}},$$

where *ρ* is the density of sample, *m* is the mass of the nuclear resonant isotope, and *D*(*E*) indicates the pDOS in the low energy region. *v*(*E*) is equal to *v*_D_ in the limit when energy (*E*) approaches zero. The pDOS shown in Fig. 2a are scaled by this Eq. (2). For a single fit range, a *v*_D_ value is determined from the fitting using an empirical power law model (Eq. (1)) of the phonon dispersion curve, noting that ‘Debye-like’ dispersion is accommodated in this form. To determine a PDF for *v*_D_, binning of hundreds of possible fit ranges was used (Table S1). The reported *v*_D_ and its uncertainty is defined as the peak position and FWHM of the fitting by asymmetric function in the probability distributions, respectively (Table S1). The details of this PDF method used in determination of *v*_D_ was discussed in ref.^68^. The elastic wave velocities (*v*_P_, *v*_S_, and *v*_Φ_) are then calculated using the *K* and *ρ* determined with XRD measurements^22^ and the *v*_D_ value, via the following equations.

3 $$\frac{3}{{{v}}_{\text{D}}^{3}} = \frac{1}{{{v}}_{\text{P}}^{3}}+ \frac{2}{{{v}}_{\text{S}}^{3}},$$

4 $$\frac{{{K}}_{{S}}}{\rho} = {{v}}_{\text{P}}^{2} -\frac{4}{{3}}{{v}}_{\text{S}}^{3},$$

Here, *v*_P_, *v*_S_, and *v*_Φ_ are defined as in the following equations:

5 $${{v}}_{\text{P}} = \sqrt{\frac{ \, {{K}}_{{S}} \, + \frac{4}{{3}}{\mu}}{\rho}},$$

6 $${{v}}_{\text{S}}=\sqrt{\frac{ {\mu}}{\rho}},$$

7 $${{v}}_{\Phi} = \sqrt{{{v}}_{\text{P}}^{2} -\frac{4}{{3}}{{v}}_{\text{S}}^{2}} = \sqrt{\frac{ {{K}}_{{S}}}{\rho}},$$

The isothermal bulk modulus (*K*_*T*_) obtained from the EoSs was converted to the adiabatic bulk modulus (*K*_*S*_) by using the Grüneisen parameter (*γ*_0_ = 0.64 ± 0.05), the exponent of its volume-independent (*q* = 1.8 ± 0.3) and Debye temperature (Θ_0_ = 1485 ± 300 K) of δ-AlOOH reported by ref.^11^.

To calculate the velocities and density of the δ-Fe13 samples having natural isotopic enrichment of iron, we applied the following equations to the velocities and density determined with the ^57^Fe-enriched experimental mass:

8 $${\rho}_{\text{nat}}= {\rho}_{\text{enr}}\frac{{\text{M}}_{\text{nat}}}{{\text{M}}_{\text{enr}}}$$

9 $${{v}}_{\text{nat}} = {{v}}_{\text{enr}}\sqrt{\frac{{\text{M}}_{\text{enr}}}{{\text{M}}_{\text{nat}}}}$$

where M_nat_ amd M_enr_ are the molecular mass of the samples having natural isotopic enrichment and ^57^Fe-enrichment, respectively.

## Supplementary Information

Supplementary Information.

## Data Availability

The data that support the findings of this study are available from the corresponding author(s) upon reasonable request.

## References

[1] Karato S-I (2011). Water distribution across the mantle transition zone and its implications for global material circulation. Earth Planet. Sci. Lett..

[2] Sandu C, Lenardic A, McGovern P (2011). The effects of deep water cycling on planetary thermal evolution. J. Geophys. Res..

[3] Nakao A, Iwamori H, Nakakuki T (2016). Effects of water transportation on subduction dynamics: Roles of viscosity and density reduction. Earth Planet. Sci. Lett..

[4] Peslier AH, Schönbächler M, Busemann H, Karato S-I (2017). Water in the Earth’s interior: Distribution and origin. Space Sci. Rev..

[5] Suzuki A, Ohtani E, Kamada T (2000). A new hydrous phase δ-AlOOH synthesized at 21 GPa and 1000 °C. Phys. Chem. Miner..

[6] Sano A, Ohtani E, Kubo T, Funakoshi K-I (2004). In situ X-ray observation of decomposition of hydrous aluminum silicate AlSiO_3_OH and aluminum oxide hydroxide d-AlOOH at high pressure and temperature. J. Phys. Chem. Solids.

[7] Sano A (2008). Aluminous hydrous mineral δ-AlOOH as a carrier of hydrogen into the core-mantle boundary. Geophys. Res. Lett..

[8] Pamato MG (2015). Lower-mantle water reservoir implied by the extreme stability of a hydrous aluminosilicate. Nat. Geosci..

[9] Fukuyama K, Ohtani E, Shibazaki Y, Kagi H, Suzuki A (2017). Stability field of phase Egg, AlSiO_3_OH at high pressure and high temperature: Possible water reservoir in mantle transition zone. J. Miner. Petrol. Sci..

[10] Abe R (2018). In situ X-ray diffraction studies of hydrous aluminosilicate at high pressure and temperature. J. Miner. Petrol. Sci..

[11] Duan Y (2018). Phase stability and thermal equation of state of δ-AlOOH: Implication for water transportation to the deep lower mantle. Earth Planet. Sci. Lett..

[12] Piet (2020). Dehydration of δ-AlOOH in Earth’s deep lower mantle. Minerals.

[13] Nishi M (2014). Stability of hydrous silicate at high pressures and water transport to the deep lower mantle. Nat. Geosci..

[14] Ohira I (2014). Stability of a hydrous δ-phase, AlOOH-MgSiO_2_(OH)_2_, and a mechanism for water transport into the base of lower mantle. Earth Planet. Sci. Lett..

[15] Walter MJ (2015). The stability of hydrous silicates in Earth's lower mantle: Experimental constraints from the systems MgO–SiO_2_–H_2_O and MgO–Al_2_O_3_–SiO_2_–H_2_O. Chem. Geol..

[16] Nishi M, Irifune T, Gréaux S, Tange Y, Higo Y (2015). Phase transitions of serpentine in the lower mantle. Phys. Earth Planet. Inter..

[17] Liu X, Matsukage KN, Nishihara Y, Suzuki T, Takahashi E (2019). Stability of the hydrous phases of Al-rich phase D and Al-rich phase H in deep subducted oceanic crust. Am. Mineral..

[18] Yuan H (2019). Stability of Fe-bearing hydrous phases and element partitioning in the system MgO–Al_2_O_3_–Fe_2_O_3_–SiO_2_–H_2_O in Earth’s lowermost mantle. Earth Planet. Sci. Lett..

[19] Nishi M (2019). Solid solution and compression behavior of hydroxides in the lower mantle. J. Geophys. Res. Solid Earth.

[20] Mashino I, Murakami M, Ohtani E (2016). Sound velocities of δ-AlOOH up to core-mantle boundary pressures with implications for the seismic anomalies in the deep mantle. J. Geophys. Res..

[21] Tsuchiya J, Tsuchiya T (2009). Elastic properties of δ-AlOOH under pressure: First principles investigation. Phys. Earth Planet. Inter..

[22] Ohira I (2019). Compressional behavior and spin state of δ-(Al, Fe)OOH at high pressures. Am. Miner..

[23] Su X (2021). The effect of iron on the sound velocities of δ-AlOOH up to 135 GPa. Geosci. Front..

[24] Tsuchiya J, Tsuchiya T, Tsuneyuki S, Yamanaka T (2002). First principles calculation of a high-pressure hydrous phase, δ-AlOOH. Geophys. Res. Lett..

[25] Sano-Furukawa A (2018). Direct observation of symmetrization of hydrogen bond in δ-AlOOH under mantle conditions using neutron diffraction. Sci. Rep..

[26] Thompson EC, Campbell AJ, Tsuchiya J (2017). Elasticity of ε-FeOOH: Seismic implications for Earth’s lower mantle. J. Geophys. Res..

[27] Masters, G., Laske, G., Bolton, H., & Dziewonski, A. The relative behavior of shear velocity, bulk sound speed, and compressional velocity in the mantle: implications for chemical and thermal structure. in S. I. Karato, A. Forte, R. Liebermann, G. Masters, & L. Stixrude (Eds.), *Earth's Deep Interior: Mineral Physics and Tomography From the Atomic to the Global Scale*. *American geophysical Union Monograph*. **117**, 63–87 (2000).

[28] Trampert J, Deschamps F, Resovsky J, Yuen D (2004). Probabilistic tomography maps chemical heterogeneities throughout the lower mantle. Science.

[29] Sturhahn, W. PHOENIX (PHOnon Excitation by Nuclear Inelastic X-ray scattering) and MINUTI (MINeral physics UTIlities) open source software. Online report, https://www.nrixs.com (2020).

[30] Zhang D (2013). Elasticity and lattice dynamics of enstatite at high pressure. J. Geophys. Res..

[31] Wicks JK, Jackson JM, Sturhahn W, Zhang D (2017). Sound velocity and density of magnesiowüstites: Implications for ultralow-velocity zone topography. Geophys. Res. Lett..

[32] Tsuchiya J, Tsuchiya T, Wentzcovitch RM (2008). Vibrational properties of δ-AlOOH under pressure. Am. Miner..

[33] Morrison RA, Jackson JM, Sturhahn W, Zhao J, Toellner TS (2019). High pressure thermoelasticity and sound velocities of Fe-Ni-Si alloys. Phys. Earth Planet. Inter..

[34] Marquardt H, Speziale S, Reichmann HJ, Frost DJ, Schilling FR (2009). Single-crystal elasticity of (Mg_0.9_Fe_0.1_)O to 81 GPa. Earth Planet. Sci. Lett..

[35] Yang J, Tong X, Lin J-F, Okuchi T, Tomioka N (2015). Elasticity of ferropericlase across the spin crossover in the Earth’s lower mantle. Sci. Rep..

[36] Dziewonski AM, Anderson DL (1981). Preliminary reference Earth model. Phys. Earth Planet. Inter..

[37] Wu Y (2016). Spin transition of ferric iron in the NAL phase: Implications for the seismic heterogeneities of subducted slabs in the lower mantle. Earth Planet. Sci. Lett..

[38] Hsu H (2017). First-principles study of iron spin crossover in the new hexagonal aluminous phase. Phys. Rev. B.

[39] Ricard, Y., Mattern, E., & Matas, J. Synthetic Tomographic Images of Slabs from Mineral Physics. in R. D. van der Hilst, J. D. Bass, J. Matas, & J. Trampert (Eds.) *Earth's Deep Mantle: Structure, Composition, and Evolution*. *American geophysical Union Monograph* **160**, 283–300 (2005).

[40] Kirby SH, Stein S, Okal EA, Rubie DC (1996). Metastable mantle phase transformations and deep earthquakes in subducting oceanic lithosphere. Rev. Geophys..

[41] Sturhahn W, Jackson JM, Lin J-F (2005). The spin state of iron in minerals of Earth’s lower mantle. Geophys. Res. Lett..

[42] Fu S (2018). Abnormal elasticity of Fe-bearing bridgmanite in the Earth’s lower mantle. Geophys. Res. Lett..

[43] Buchen J (2018). Equation of state of polycrystalline stishovite across the tetragonal-orthorhombic phase transition. J. Geophys. Res. Solid Earth.

[44] Deuss, A., Andrews, J., & Day, E. Seismic observations of mantle discontinuities and their mineralogical and dynamical interpretation. In *Physics and Chemistry of the Deep Earth* (Ed. Karato, S.-i.) pp. 297–323 (Wiley, 2013).

[45] Fukao Y, Obayashi M (2013). Subducted slabs stagnant above, penetrating through, and trapped below the 660 km discontinuity. J. Geophys. Res. Solid Earth.

[46] Birch F (1961). Composition of the Earth’s mantle. Geophys. J. Int..

[47] Garnero EJ, McNamara AK, Shim S-H (2016). Continent-sized anomalous zones with low seismic velocity at the base of Earth’s mantle. Nat. Geosci..

[48] Brown JM, Shankland TJ (1981). Thermodynamic parameters in the Earth as determined from seismic profiles. Geophys. J. R. astr. Soc..

[49] Wentzcovitch RM, Wu Z, Carrier P (2010). First principles quasiharmonic thermoelasticity of mantle minerals. Rev. Miner. Geochem..

[50] Liu J (2017). Hydrogen-bearing iron peroxide and the origin of ultralow-velocity zones. Nature.

[51] Song Y, He K, Sun J, Ma C, Wan M, Wang Q, Chen Q (2019). Effects of iron spin transition on the electronic structure, thermal expansivity and lattice thermal conductivity of ferropericlase: A first principles study. Sci. Rep..

[52] Koelemeijer P, Deuss A, Ritsema J (2017). Density structure of Earth’s lowermost mantle from Stoneley mode splitting observations. Nat. Commun..

[53] Lau HCP (2017). Tidal tomography constrains Earth’s deep-mantle buoyancy. Nature.

[54] Burke K, Steinberger B, Torsvik TH, Smethurst MA (2008). Plume generation zones at the margins of large low shear velocity provinces on the core–mantle boundary. Earth Planet. Sci. Lett..

[55] Niu Y (2018). Origin of the LLSVPs at the base of the mantle is a consequence of plate tectonics: A petrological and geochemical perspective. Geosci. Front..

[56] Helffrich GR, Wood BJ (2001). The Earth’s mantle. Nature.

[57] van Keken PE, Hacker BR, Syracuse EM, Abers GA (2011). Subduction factory: 4. Depth-dependent flux of H2O from subducting slabs worldwide. J. Geophys. Res..

[58] Ohtani E (2020). The role of water in Earth’s mantle. Natl. Sci. Rev..

[59] Tan E, Gurnis M (2005). Metastable superplumes and mantle compressibility. Geophys. Res. Lett..

[60] Wolf AS, Jackson JM, Dera P, Prakapenka VB (2015). The thermal equation of state of (Mg, Fe)SiO_3_ bridgmanite (perovskite) and implications for lower mantle structures. J. Geophys. Res. Solid Earth.

[61] Thomson AR (2019). Seismic velocities of CaSiO_3_ perovskite can explain LLSVPs in Earth’s lower mantle. Nature.

[62] Kawazoe T (2017). Single crystal synthesis of δ-(Al, Fe)OOH. Am. Miner..

[63] Toellner TS (2000). Monochromatization of synchrotron radiation for nuclear resonant scattering experiments. Hyperfine Interact..

[64] Sturhahn, W., & Jackson, J. M. Geophysical applications of nuclear resonant spectroscopy. in E. Ohtani (Ed.) *Advances in High-Pressure Mineralogy*, *Geological Society of America Special Paper*, **421**, 157–174 (2007).

[65] Dewaele A, Torrent M, Loubeyre P, Mezouar M (2008). Compression curves of transition metals in the Mbar range: Experiments and projector augmented-wave calculations. Phys. Rev. B.

[66] Chijioke AD, Nellis WJ, Soldatov A, Silvera IF (2005). The ruby pressure standard to 150 GPa. J. Appl. Phys..

[67] Sturhahn W (2000). CONUSS and PHOENIX: Evaluation of nuclear resonant scattering data. Hyperfine Interact..

[68] Sturhahn W (2004). Nuclear resonant spectroscopy. J. Phys. Condens. Matter.

