# Supplementary material for: The influence of δ-(Al,Fe)OOH on seismic heterogeneities in Earth’s lower mantle
Source: Sci Rep. 2021 Jun 8;11:12036. doi: 10.1038/s41598-021-91180-9 (PMC8187711; doi:10.1038/s41598-021-91180-9)
Supplement: Supplementary file 1 — Supplementary Information. [file 41598_2021_91180_MOESM1_ESM.pdf]

Supplementary Information

The influence of δ-(Al,Fe)OOH on seismic heterogeneities in Earth's lower mantle

Itaru Ohira, Jennifer M. Jackson, Wolfgang Sturhahn, Gregory J. Finkelstein, Takaaki Kawazoe,
Thomas S. Toellner, Akio Suzuki, and Eiji Ohtani

Supplementary Figures

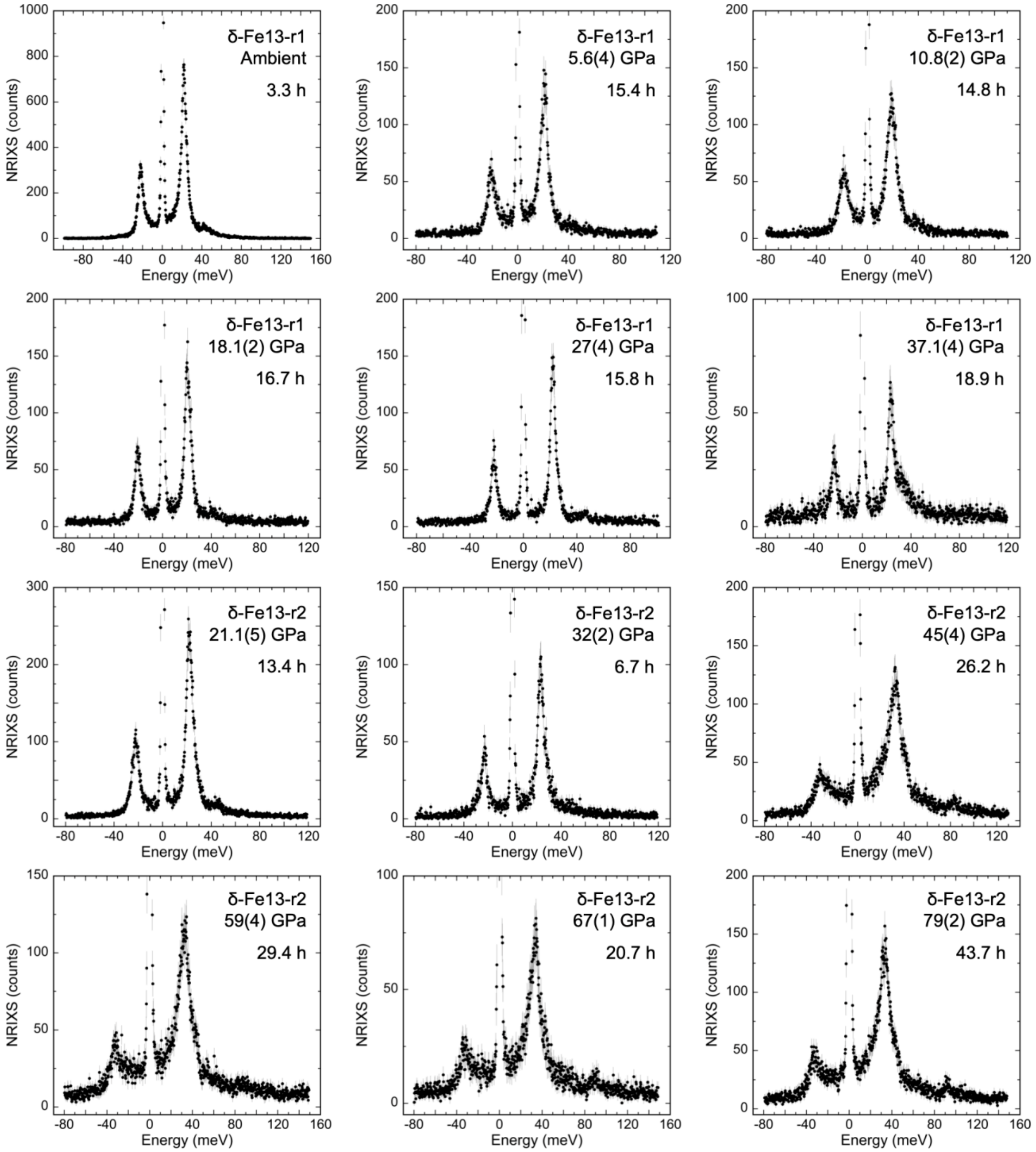

Fig. S1 Raw (unnormalized) NRIXS spectra for the δ-Fe13 samples. The energy step-size is 0.25 meV. Uncertainties in pressure are given in parentheses for the last significant digit. The numbers below the run numbers and pressure values in each panel indicate the collection time (hours).

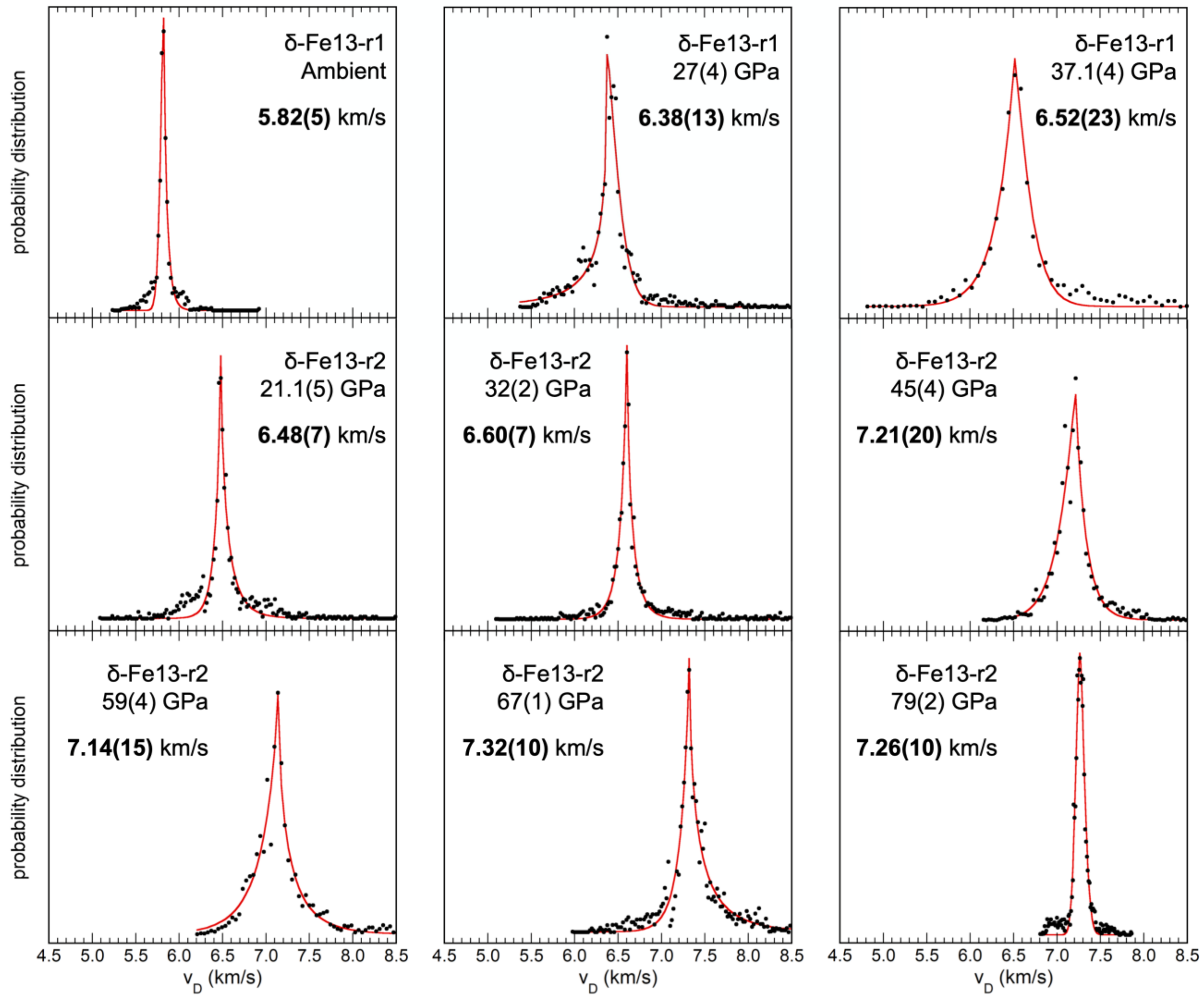

Fig. S2 The probability distribution functions (PDFs) of δ-Fe13 at 9 experimental conditions. To demonstrate the v_D shift of the probability distribution, all of the horizontal axes are fixed at same range (4.5 to 8.5 km/s). The ^{57}Fe concentration of the sample (96.64 % ^{57}Fe) was used. Uncertainties are given in parentheses for the last significant digit(s). The properties of the PDFs are listed in Table S1.

Supplementary Tables

Table S1. Properties of the probability distribution function (PDF) for each reported Debye sound velocity (v_D).

						Properties of v_D PDF						v_D distribution fit[b]	
Run/Sample	Pressure	E_{min}[a]	E_{max}[a]	Number of fits to pDOS fit		Number of bins	Bin size	Ave. v_D	RMS of v_D	Skewness	Kurtosis	Fitted v_D	v_D error
	(GPa)	(meV)	(meV)	Successful	Failed		(km/s)	(km/s)	(km/s)			(km/s)	(km/s)
<u>run 1</u>	0.0001	2.7	14.7	980	10	84	0.020	5.82	0.12	1.03	13.62	5.82	0.05
δ-Fe13-r1	27(4)	3.8	18.8	1553	43	223	0.025	6.43	0.41	3.46	27.46	6.38	0.13
Fe# = 0.127(3)	37.1(4)	4.7	18.7	1346	32	114	0.071	6.88	1.29	3.49	15.32	6.52	0.23
<u>run 2</u>	21.1(5)	1.7	17.7	1776	54	208	0.020	6.53	0.37	2.14	15.09	6.48	0.07
δ-Fe13-r2	32(2)	1.7	17.7	1792	38	191	0.020	6.66	0.35	2.28	13.62	6.60	0.07
Fe# = 0.133(3)	45(4)	3.7	20.7	2005	75	101	0.030	7.20	0.29	1.99	13.03	7.21	0.20
	59(4)	3.7	21.7	2139	207	115	0.040	7.20	0.44	2.64	14.80	7.14	0.15
	67(1)	6.2	22.5	1753	138	337	0.020	7.42	0.50	3.29	24.37	7.32	0.10
	79(2)	3.7	22.7	2628	0	105	0.010	7.26	0.12	-0.08	7.47	7.26	0.10

[a] The minimum (E_{min}) and maximum (E_{max}) energy represent bounds in determining the v_D from fits using the power law model (Eq. 1 in the main text) to the pDOS, noting that 'Debye-like' dispersion is accommodated in this form. Fits are performed at a range of E_{min} and E_{max} values within these bounds for hundreds of fits to the pDOS and these results populate the PDF for each reported v_D. Fits are not performed below E_{min} or above E_{max}.

[b] The reported values in this study. These values are obtained from fitting an asymmetric function to the PDFs and used in the calculation of elastic wave velocities (Table S2).

Table S2. Experimental data of δ-Fe13.

		^{57}Fe enriched			Natural Fe abundance[c]							
Run/Sample	Pressure (GPa)	Density[a] (g/cm^3)	f_{LM}	v_D[b] (km/s)	Density (g/cm^3)	v_D (km/s)	v_P (km/s)	v_S (km/s)	v_Φ (km/s)	v_S/v_Φ	K_S (GPa)	G (GPa)
<u>run 1</u>	0.0001	3.747(1)	0.814(1)	5.82(5)	3.739(1)	5.83(5)	8.74(4)	5.27(4)	6.27(2)	0.84(1)	147(1)	104(2)
δ-Fe13-r1	27(4)	4.220(1)	0.814(5)	6.38(13)	4.211(1)	6.38(15)	11.10(20)	5.70(14)	8.93(23)	0.64(3)	336(6)	137(7)
Fe# = 0.127(3)	37.1(4)	4.419(6)	0.864(6)	6.52(23)	4.409(6)	6.52(23)	9.66(26)	5.91(22)	6.84(28)	0.86(7)	206(17)	154(11)
<u>run 2</u>	21.1(5)	4.180(1)	0.826(3)	6.48(7)	4.171(1)	6.48(7)	10.85(6)	5.81(6)	8.52(6)	0.68(1)	303(4)	141(3)
δ-Fe13-r2	32(2)	4.324(2)	0.849(4)	6.60(7)	4.314(2)	6.61(7)	10.76(18)	5.93(6)	8.30(24)	0.71(3)	297(17)	152(3)
Fe# = 0.133(3)	45(4)	4.562(3)	0.904(2)	7.21(20)	4.551(3)	7.21(20)	12.05(17)	6.47(18)	9.46(14)	0.68(3)	407(12)	190(11)
	59(4)	4.711(5)	0.907(3)	7.14(15)	4.701(5)	7.14(15)	12.33(15)	6.38(14)	9.88(15)	0.65(2)	459(14)	192(8)
	67(1)	4.792(7)	0.907(3)	7.32(10)	4.781(7)	7.33(10)	12.61(14)	6.55(10)	10.09(16)	0.65(2)	487(15)	205(6)
	79(2)	4.906(11)	0.916(2)	7.26(10)	4.895(11)	7.27(10)	12.81(14)	6.49(10)	10.40(15)	0.62(2)	529(15)	206(6)

[a] Determined using the sample compositions with 96.64% concentration of ^{57}Fe, and the spin crossover equation of state of δ-($Al_{0.877}{}^{57}Fe_{0.123}$)OOH sample (δ-Fe12) calculated with one hydrogen atom in the chemical formula, reported by ref. 1 and the Mie-Grüneisen parameters of δ-AlOOH reported by ref. 2.

[b] Determined by the probability distribution function method.

[c] Calculated from values corresponding to ^{57}Fe enriched mass to those corresponding to natural isotopic enrichment using the Eqs. (8) and (9) in the main text. Fe# = Fe/(Al+Fe). The numbers in parentheses are uncertainties in the last significant digit(s).

Table S3. The velocity profiles of the δ-Fe13 samples at room temperature and the mantle geotherm and PREM.

			PREM			δ-Fe13 at room temperature			δ-Fe13 under the mantle geotherm[a]		
Depth	P	T	v_P	v_S	v_Φ	v_P[b]	v_S[c]	v_Φ[d]	v_P[e]	v_S[f]	v_Φ[d]
(km)	(GPa)	(K)		(km/s)			(km/s)			(km/s)	
1871	80.37	2225	12.67	6.87	9.87	12.85(18)	6.51(18)	10.42(15)	12.59(22)	6.32(22)	10.26(20)
1971	85.43	2249	12.78	6.92	9.98	12.96(18)	6.52(18)	10.54(15)	12.69(22)	6.33(22)	10.38(19)
2071	90.56	2273	12.90	6.97	10.09	13.06(18)	6.54(18)	10.66(15)	12.80(22)	6.34(22)	10.50(19)
2171	95.76	2296	13.02	7.01	10.19	13.16(18)	6.55(18)	10.77(15)	12.91(23)	6.35(22)	10.62(19)
2271	101.04	2319	13.13	7.06	10.30	13.27(19)	6.57(18)	10.88(15)	13.01(23)	6.37(22)	10.73(19)
2371	106.39	2341	13.25	7.10	10.40	13.37(19)	6.59(18)	11.00(15)	13.11(23)	6.38(22)	10.85(19)
2471	111.82	2363	13.36	7.14	10.51	13.47(19)	6.60(18)	11.11(15)	13.22(23)	6.39(22)	10.96(19)
2571	117.35	2384	13.48	7.19	10.62	13.57(19)	6.62(18)	11.22(15)	13.32(23)	6.41(22)	11.07(19)
2671	122.97	2405	13.60	7.23	10.73	13.67(19)	6.63(19)	11.33(15)	13.42(23)	6.42(22)	11.19(18)
2771	128.71	2426	13.69	7.27	10.81	13.78(19)	6.65(19)	11.43(15)	13.53(23)	6.44(22)	11.30(18)
2871	134.56	2446	13.71	7.26	10.85	13.88(19)	6.67(19)	11.54(15)	13.63(23)	6.45(22)	11.41(18)

[a] The mantle geotherm reported by ref. 3.

[b] Extrapolated values by using a linear fitting (Birch's law, ref. 4) on the measured v_P and the density calculated from the EoS of the δ-Fe12 (ref. 1) with the Mie-Grüneisen parameters of δ-AlOOH (ref. 2) using MINUTI software (Sturhahn, W. Online report, https://www.nrixs.com) at 45 GPa, 59 GPa, 67 GPa, and 79 GPa.

[c] Calculated from v_P and v_Φ at room temperature (298 K) and the Eqs. (5)–(7) in the main text.

[d] Determined by the EoS of the δ-Fe12 (ref. 1) with the Mie-Grüneisen parameters of δ-AlOOH (ref. 2) using MINUTI software.

[e] Calculated from v_S and v_Φ at the mantle geotherm (ref. 3) and the Eqs. (5)–(7) in the main text.

[f] Calculated by using the value of $dv_S/dT = 1\times10^{-4}$ (km/s)K^{-1} and the temperatures reported by ref. 3.

The numbers in parentheses are uncertainties in the last significant digit(s).

Supplementary Information References

1. Ohira, I. et al. Compressional behavior and spin state of δ-(Al,Fe)OOH at high pressures. *Am. Mineral.* **104**, 1273–1284 (2019).

2. Duan, Y. et al. Phase stability and thermal equation of state of δ-AlOOH: Implication for water transportation to the Deep Lower Mantle. *Earth Planet. Sci. Lett.* **494**, 92–98 (2018).

3. Brown J. M. & Shankland T. J. Thermodynamic parameters in the Earth as determined from seismic profiles. *Geophys. J. R. astr. Soc.* **66**, 579–596 (1981).

4. Birch, F. Composition of the Earth's mantle. *Geophys. J. Int.* **4**, 295–311 (1961).
